# Chicken Coccidiosis in Peri-Urban Family Farming in Two South American Countries: Prevalence and Circulating *Eimeria* spp.

**DOI:** 10.3390/ani15070982

**Published:** 2025-03-29

**Authors:** Mariela L. Tomazic, Jesica D. Britez, María Luz Pisón-Martínez, Pablo Barbano, Zulma Canet, Marcos D. Trangoni, Tomás J. Poklepovich, Facundo Cubas, Raúl Alegría-Morán, Galia Ramírez-Toloza, Anabel E. Rodríguez

**Affiliations:** 1 Instituto de Patobiología Veterinaria (IPVET), Unidad de Doble Dependencia, INTA-CONICET, Hurlingham B1686, Argentina; britez.jesica@inta.gob.ar (J.D.B.); pison.maria@inta.gob.ar (M.L.P.-M.); 2 Cátedra de Biotecnología, Facultad de Farmacia y Bioquímica (FFyB), Universidad de Buenos Aires (UBA), Buenos Aires C1113, Argentina; 3 Cátedra de Parasitología, Facultad de Ciencias Veterinarias, Universidad de Buenos Aires (UBA), Buenos Aires C1113, Argentina; 4 Estación Experimental Agropecuaria (EEA), INTA, Luján B6700, Argentina; 5 EEA, INTA Pergamino, Buenos Aires C1113, Argentina; canet.zulma@inta.gob.ar; 6 Instituto de Agrobiotecnología y Biología Molecular (IABIMO), INTA-CONICET, Hurlingham B1686, Argentina; trangoni.marcos@inta.gob.ar; 7 Unidad Operativa Centro Nacional de Genómica y Bioinformática-ANLIS “Dr. Carlos G. Malbrán”, Buenos Aires C1113, Argentina; tcaride@gmail.com (T.J.P.); facundogcuba@gmail.com (F.C.); 8 Cátedra Física, FFyB, Universidad de Buenos Aires (UBA), Buenos Aires C1113, Argentina; 9 Escuela de Medicina Veterinaria, Sede Santiago, Facultad de Recursos Naturales y Medicina Veterinaria, Universidad Santo Tomás, Santiago 8370003, Chile; ralegria2@santotomas.cl; 10 Laboratorio de Parasitología y Enfermedades Parasitarias, Facultad de Ciencias Veterinarias y Pecuarias, Universidad de Chile, Santa Rosa 11735, La Pintana, Santiago 8820808, Chile; galiaram@uchile.cl; 11 Instituto de Patobiología (IP), IP-IPVET, Hurlingham B1686, Argentina

**Keywords:** family farming, poultry, chicken coccidiosis, *Eimeria* sp., sulfonamides, One Health

## Abstract

Family poultry production systems (FPPSs) in Chile and Argentina produce high-quality and nutritious food. However, little is known about chicken coccidiosis in these production systems. This work aimed to determine the *Eimeria* sp. positivity rate, circulating species, general farm management, and knowledge of the disease in FPPSs by gaining access to peri-urban markets in these two countries. The overall *Eimeria* sp. positivity rate, determined in 88 fecal samples, was 85.1%. Oocysts per gram in Argentinean meat-producing FPPSs were significantly higher than in Argentinean and Chilean egg-producing FPPSs. Multiplex-PCR based on seven *Eimeria* spp. Sequence-Characterized Amplified Region (SCAR) markers showed a great diversity of circulating species, with *E. mitis* (70.3%), *E. acervulina* (62.2%), and *E. tenella* (59.5%), followed by *E. maxima* (43.2%), *E. praecox* (32.4%), *E. necatrix* (18.9%), and *E. brunetti* (5.4%). Low awareness among family producers and low implementation of control measures were found. Importantly, pathogenic species were found even in asymptomatic chickens, which represents a potential chicken health threat. Furthermore, the administration of sulfonamides to broilers with clinical signs poses a risk of environmental contamination. This is the first comprehensive cross-sectional study showing that *Eimeria* sp. is a persistent parasite in peri-urban FPPSs in Argentina and Chile.

## Simple Summary

The relevance of coccidiosis, a parasitic disease of chickens, in family poultry production systems (FPPSs) was assessed in Buenos Aires, Argentina, and Santiago, Chile, by analyzing samples obtained from chicken pens in the laboratory, and by a survey addressed to producers. We found that coccidiosis is a prevalent disease in FPPSs, disseminating parasitic species of these coccidia in the environment. We also estimated a low awareness of coccidiosis and its association with other diseases and a lack of prevention strategies to avoid outbreaks among family producers. This descriptive study is the first conducted in FPPSs in this region. Its findings can help the introduction of sanitary improvements to attenuate the parasite’s dissemination and improve chicken health, as well as aid in diminishing the use of chemical anticoccidials, contributing to food safety and a minimal environmental impact of anticoccidials used in poultry production.

## 1. Introduction

Family farming (FF) is key to food security in low- and middle-income countries, providing high-quality food and diversified products. In Argentina and Chile, FF is a highly representative production system that mainly involves rural women and offers an opportunity for economic independence [1]. Family poultry production systems (FPPSs) in developing countries include a variety of small-scale production strategies in rural, urban, and peri-urban areas. Four broad categories can be distinguished: small-scale extensive scavenging, extensive, semi-intensive, and small-scale intensive, with different characteristics and flock sizes [2].

Chicken meat and eggs are an important source of high-quality nutrition for humanity worldwide [3]. Poultry production continuously increases, and sustainable systems embracing the One Health approach are needed.

In this context, chicken coccidiosis is one of the most important parasitic diseases affecting chicken health, reducing performance and productivity. Control measures in FPPSs are mainly based on good husbandry practices and biosecurity measures, along with the use of anticoccidials, usually added to feed or water. The latter are being limited due to their environmental impact and the emergence of eimerian-resistant strains. The high cost of anticoccidial vaccines limits their application in FPPSs [4,5].

The global economic impact of coccidiosis on the industry has been estimated at around USD 14.5 billion [6], including direct and indirect costs such as prophylaxis and treatment.

Seven chicken *Eimeria* spp., *E. acervulina*, *E. brunetti*, *E. maxima*, *E. mitis*, *E. necatrix*, *E. praecox*, and *E. tenella*, were described several decades ago, with different levels of pathogenicity, and their occurrence and distribution vary geographically and between types of production systems [7,8,9,10,11,12,13]. Mixed infections are common, but single infections have also been reported [10]. In addition to these seven chicken species, new genetic variants, named *E. lata*, *E. nagambie*, and *E. zaria*, have recently been described with a worldwide distribution [12,14,15,16].

Little is known about the impact of chicken coccidiosis on FPPSs in Argentina and Chile. The current work aimed to assess the prevalence of *Eimeria* sp. and to identify the seven circulating species, to improve the knowledge on chicken coccidiosis in FPPSs by gaining access to markets in four peri-urban regions of similar latitudes of both countries. Management factors were also identified, to reveal the state of the art of chicken coccidiosis in FPPSs. This constitutes the first comprehensive study of chicken coccidiosis in the region. It provides a deeper understanding of the disease, which is essential for implementing better control strategies to prevent future outbreaks in these production systems.

## 2. Materials and Methods

### 2.1. Study Area and Production Systems

**Figure 1:**
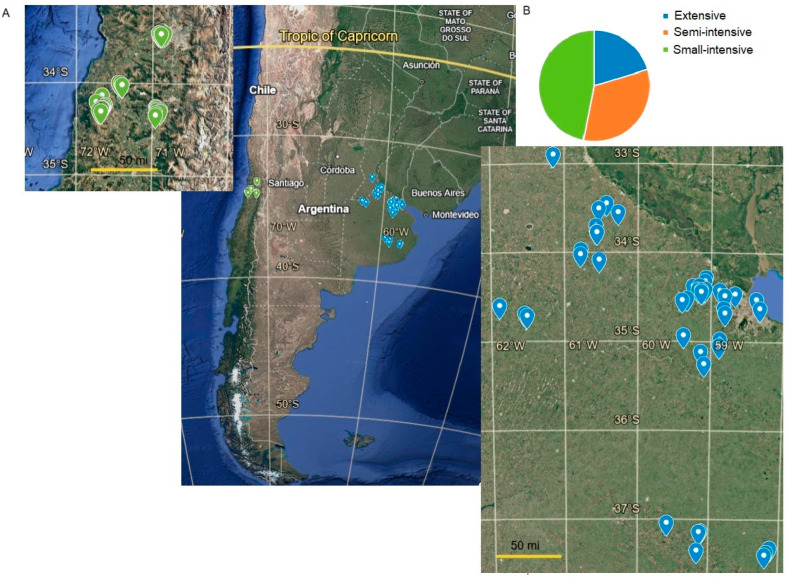
Geo-localization and categories of FPPSs. (**A**). Geo-localization of studied farms in Argentina (blue) and Chile (green). (**B**). Proportion of categories of FPPSs according to the FAO (2014) [2].

A total of 88 family poultry farms with access to local markets were included in this study. Argentine FPPSs comprised meat (MP) (*n* = 12), eggs (EP) (*n* = 32), and mixed production (*n* = 5), while Chilean FPPSs included only EP (*n* = 39) farms, where chickens were reared on the floor. Farms were located in the peri-urban regions of the Metropolitan Area of Buenos Aires (AMBA), Argentina; Province of Buenos Aires (BA), Argentina; Santiago Metropolitan Region (RM), Chile; and Libertador General Bernardo O’Higgins Region (OH), Chile. The regions under study were geo-located between the latitudes of −33.0000 and −37.5000 in both countries (Figure 1A). FPPSs were chosen by convenience and each owner agreed to this study by signing an informed consent form.

### 2.2. Sampling and Collection Instrument

Sampling was performed during spring and fall 2023, by collecting duplicate samples of at least 10–12 fresh fecal pellets (≥25 mg each) from each shed, which approximately corresponds to the deposition of 10% of the average number of chickens per shed. Collection was carried out following a ‘W’ route through the unit, starting from a corner, with a sample collected every two to five steps. In the case of mixed FPPSs, samples were separately collected from sheds devoted to egg and broiler production and analyzed with the other EP or MP samples. Thus, the total number of collected samples was 93 from 88 FPPSs. Samples were collected in plastic bags, stored at 4 °C, and immediately processed in no longer than seven days. Animals were not manipulated, and only fecal samples collected from the floor were included in this study.

Additionally, a survey of FPPS owners (supplementary) was carried out through a Google form and covered aspects related to known risk factors and coccidiosis, including (i) cleaning and disinfection of pens, (ii) use of chemicals and anticoccidials, (iii) knowledge of the disease, and (iv) observed signs of coccidiosis.

### 2.3. Eimeria-Oocysts Quantification and Isolation

*Eimeria* sp. was quantified by counting the oocysts per gram (OPG) of fecal matter using the modified McMaster test in a 4-chamber slide. Briefly, 5 mg of feces was homogenized in a mortar in saturated saline solution, filtered through a metal strainer, and stirred for 5 min. The McMaster was filled with the saline suspension (0.5 mL/chamber) and allowed to rest for 5 min before counting. Floating *Eimeria* sp. oocysts were microscopically recognized by their morphology following established procedures [17,18]. OPG levels were categorized as negative (0), low (<1800), medium (1801–6000), or high (>6000) [19]. These assigned categories were confirmed in the duplicate samples taken from each shed. Eggs of gastrointestinal helminths were recorded when observed microscopically in the McMaster chamber.

*Eimeria* sp. oocysts were then isolated from positive samples by flotation in a saturated sodium chloride solution (D = 1.2 g/L) and allowed to sporulate by incubation for 48 to 72 h in 2% potassium dichromate at 28 °C in a humid atmosphere, under shaking. Sporulation was confirmed by microscopic observation, and oocysts were counted in a Neubauer chamber (NB) and stored at 4 °C until usage.

### 2.4. Molecular Identification of Eimeria sp. and Sequencing

Isolated *Eimeria* sp. oocysts (*n* ≥ 10^5^) were washed with sterile deionized water and bleached with sodium hypochlorite solution for 10 min at 4 °C. Then, they were ruptured using acid-washed 425–600 μm glass beads (Sigma, Livonia, MI, USA) in a Genie cell disruptor (Scientific Industries, Inc., Bohemia, NY, USA) (6 cycles of 2 min at 2700 rpm). Between each cycle, samples were cooled at 0 °C for 2 min. Subsequently, DNA was extracted using the Wizard^®^ Genomic DNA Purification Kit (Promega, Road Madison, WI, USA), and the concentration and quality of the DNA were assessed in a P-Class P330 Nanophotometer (Implen, Munich, Germany). Molecular species identification was carried out by two multiplex PCR (m-PCR) protocols in each sample, with some modifications [20]. Briefly, m-PCR was divided into two reactions (M1 and M2), where M1 contained 1X GoTaq Buffer (Promega, Road Madison, WI, USA), 2.5 U GoTaq polymerase (Promega, Road Madison, WI, USA), 200 µM dNTPs (Promega, Road Madison, WI, USA), and 0.72, 0.56, and 0.56 µM RAPD-SCAR specific oligonucleotides, as previously published [20,21], of *E. acervulina*, *E. maxima*, and *E. tenella*, respectively. The M2 reaction mixture contained the same amounts of buffer, GoTaq polymerase, and dNTPs, and 0.88, 0.72, 0.56, and 0.56 µM of RAPD-SCAR oligonucleotides of *E. brunetti*, *E. mitis*, *E. necatrix*, and *E. praecox*, respectively. M-PCR products of *E. acervulina* (812 bp), *E. tenella* (520 bp), and *E. maxima* (272 bp) in M1, and *E. brunetti* (620 bp), *E. mitis* (460 bp), *E. praecox* (354 bp), and *E. necatrix* (200 bp) in M2 were separated by electrophoresis on 2.5% agarose gels stained with 1X Sybr Safe DNA gel stain (ThermoFisher Scientific), then revealed under UV light in a Gel Doc Go Gel Imaging System (Biorad, Biorad, CA, USA).

To generate plasmids containing each of the 7 RAPD-SCAR fragments, single PCRs were carried out using DNA extracted from an aliquot of the Prevencoc E7 live vaccine (Inmuner^®^, Concepción del Uruguay, Argentina), which contains oocysts of the 7 chicken *Eimeria* spp. as a template. Amplicons were then cloned into pGEM^®^-T (Promega, Road Madison, WI, USA) and used to transform *E. coli* competent cells. *Eimeria* sp.-SCAR-positive clones were selected and grown in LB-ampicillin, and plasmids were purified using the Wizard^®^ Plus SV Miniprep kit (Promega, Road Madison, WI, USA) and quantified by Nanodrop. A mix containing 10 µg of each purified plasmid was prepared and used in a 1/1000 dilution as a positive control for m-PCR.

Additionally, six out of the seven chicken *Eimeria* sp. amplified from 1 to 3 isolates were subjected to Next-Generation Sequencing Technology. Briefly, the PCR products were mixed and underwent a tagmentation step during library preparation. This process produced homogeneous fragments that were subsequently sequenced, mapped, and assembled as part of the bioinformatic analysis. Libraries were generated using the DNA Prep (Illumina, San Diego, CA, USA), and they were sequenced Paired-End 2 × 150 pb in an Illumina MiSeq. The bioinformatic analysis consisted of two main steps. The first step involved quality control of the reads obtained from sequencing, using FastQC to assess the overall quality of the reads both before and after trimming, Trim Galore to trim adapter sequences and perform quality trimming, and Kraken2 to check for potential contamination. The second step involved mapping the reads and generating a consensus sequence using an in-house script, which included the following tools: (i) bowtie2 to map the raw reads to the RAPD-SCAR markers’ genomic sequences Ac-A03-811 (AY571520.1), Br-J18-626 (AY571556.1), Mx-A09-1008 (AY571588.1), Mt-A03-460 (AY571503.1), Pr-A03-718 (AY571602.1), and Tn-K04-539 (AY571634.1), (ii) Samtools to sort BAM files and obtain mapping statistics, and (iii) iVar software (FastqQC: 0.12.1, Trim galore: 0.6.10, Kraken2: 2.1.2, bowtie2: 2.3.4.1, samtools: 1.19, ivar: 1.3.1) to generate consensus sequences.

Additionally, the script includes two helper modules: one to generate a readable output table summarizing BAM file statistics, and another to create visual graphics displaying the vertical depth at each position and the overall coverage for each consensus sequence. The identity of each sequenced RAPD-SCAR was verified by Nucleotide-BLAST (n-BLAST). Sequences were submitted to GenBank under accession numbers PP328761–PP328774.

### 2.5. Statistical Analysis

The statistical significance of the data was analyzed with GraphPad Prism V.8.0.1 software, using the Chi-square test or the Fisher exact test for categorical variables and the Mann–Whitney test for continuous variables.

## 3. Results

### 3.1. Family Poultry Production Systems

Farms with access to local markets were located in the peri-urban areas of the provinces of Buenos Aires, Argentina, and Santiago, Chile (Figure 1A). In the former, 25% had broilers for meat production (MP), 65% had laying hens for egg production (EP), and 10% had sheds for both kinds of production. Meanwhile, 100% of the farms in Chile had laying hens (Table S1).

Of the 88 smallholder farms visited, 7% used agroecological management. Flock sizes varied widely between 20 and 2500 birds in both countries, with a mean of 157 and 100 birds for MP and EP, respectively. According to the characteristics of FPPSs described by the FAO (2014) [2], 20.2, 33.0, and 46.8% were categorized as extensive, semi-intensive, and small intensive, respectively (Figure 1B). None of the farmers used live *Eimeria* vaccines.

### 3.2. Eimeria Occurrence

**Figure 2:**
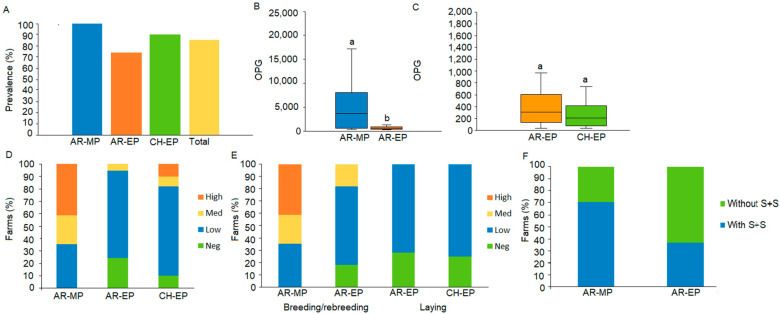
*Eimeria* sp. prevalence, oocyst quantification, and OPG levels in FPPSs. (**A**) Farm percentages of *Eimeria* sp. in Argentina meat production farms (AR-MP), egg production farms (AR-EP), and Chilean egg production farms (CH-EP). (**B**,**C**). *Eimerian*-oocyst excretion quantified by oocysts per gram (OPG) in AR-MP and AR-EP farms (**B**) and AR-EP and CH-EP farms (**C**). Different lowercase letters indicate significant differences. (**D**) Proportions of farms with different OPG levels (high, medium, low, and negative) among the production systems studied in both countries. (**E**). Proportions of farms with different OPG levels according to the production stage in AR-MP and AR-EP breeding and rebreeding stages, AR-EP and CH-EP laying stage. (**F**) Percentages of farms with or without recorded clinical signs (CS) in Argentine MP and EP.

The global prevalence of *Eimeria* sp. in chicken feces was 85.1%, standing at 100.0% in broiler (*n* = 17) and 73.7% in hen (*n* = 37) sheds in Argentina, and 89.7% in hen sheds in Chile (*n* = 39) (Figure 2A). In addition, 40.5% of the samples analyzed from Argentina and Chile contained eggs of gastrointestinal helminths.

The measured OPGs ranged from 0 to 22,946 and did not follow a normal distribution. Most farms (69.5%) showed low OPG levels (<1800), while negative, intermediate (1801–6000), and high (>6001) levels were found in 15.8, 4.2, and 10.5% of the farms, respectively. A non-parametric Mann–Whitney test showed that the OPG mean value was 6326; 438; and 940 for Argentine MP (AR-MP), Argentina EP (AR-EP), and Chilean EP farms (CH-EP), respectively. Differences between AR-MP and AR-EP were significant at 210 (*p* = 0.0023), while differences between AR-EP and CH-EP were non-significant (*p* = 0.2397) (Figure 2B,C).

As shown in Figure 2D, negative OPG values were only observed in AR-EP (24.3%) and CH-EP (10.3%), while high OPGs were found in 41.2% of AR-MP farms and 10.3% of CH-EP farms. It was also observed that 70.3% of the low OPGs were found in AR-EP, 71.8% in CH-EP, and 35.3% in AR-MP. When comparing OPG levels according to the rearing stage for each type of production, it was observed that while high OPG levels were more frequent than low ones in broilers at the breeding and rebreeding stages (up to 45 days of age), laying hens had low OPG levels at the same production stages (up to 60 days of age) (Figure 2E). Negative OPG values were mainly found in laying hens over 5 months of age, both in Argentinean and Chilean farms (Figure 2E; Table S1).

During the survey, Argentinean veterinarians and farmers recorded clinical signs associated with coccidiosis, including diarrhea of variable intensity and an uneven chicken size. In contrast, no signs were observed or recorded in Chile. In Argentina, clinical signs were significantly more frequent (*p* = 0.025) in broilers than in laying hens (70.6% vs. 36.7% for MP and EP, respectively) (Figure 2F).

### 3.3. Molecular Analysis of Eimeria spp.

**Figure 3:**
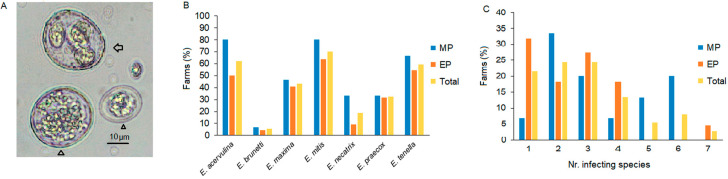
*Eimerian*-oocysts and the distribution of the number and chicken species. (**A**). Micrograph of purified oocysts of *Eimeria* spp. that were subjected to molecular identification. A sporulated (arrow) and two unsporulated (arrowhead) oocysts with different sizes are observed. Total augmentation 400×. (**B**). Farm prevalence of *Eimeria* sp. (**C**). Percentages of farms with mixed and single infections.

Thirty-seven of the eighty *Eimeria* sp.-positive samples (46.3%) were analyzed. *Eimerian*-oocysts were purified, sporulated (Figure 3A), and subjected to DNA extraction and m-PCR. The results revealed the presence of the seven defined chicken *Eimeria* spp. They showed the following farm prevalence: *E. mitis*, 70.3%; *E. acervulina*, 62.2%; *E. tenella*, 59.5%; *E. maxima*, 43.2%; *E. praecox*, 32.4%; *E. necatrix*, 18.9%; and *E. brunetti*, 5.4% (Figure 3B). No significant differences were observed (*p* = 0.9764) between MP and EP. Infections with combinations of two and three species were the most frequent (24.3 and 24.3%, respectively), followed by single infections (21.6%) and mixed infections with four, six, five, or seven species (Figure 3C).

Single infections were considerably more frequent in EP than in MP (Figure 3C). Interestingly, six out of eight single infections in EP contained *E. mitis*. Infections with mixtures of two, three, or six species were found in clinical and subclinical cases. *E. tenella* or *E. brunetti* was found in the majority (16/21) of samples with sanguinolent diarrhea, but they were also found in asymptomatic cases (6/14).

To further assess the sequence identity of the species identified by the two m-PCRs used in this work, NGT sequencing of SCAR from *E. acervulina* (*n* = 3); *E. brunetti* (*n* = 1); *E. maxima* (*n* = 2); *E. mitis* (*n* = 2); *E. praecox* (*n* = 1); and *E. tenella* (*n* = 4) was performed by mixing the amplicons from both m-PCRs per isolate and subjecting the mixes to library preparation and sequencing. Sequenced SCARs were used as queries for n-BLAST, which showed an identity percentage ranging from 98.7 to 100.0%.

### 3.4. Cleaning and Disinfection

The frequency of cleaning and the type of disinfectant used were recorded in the surveys (Table S1). While 33.3% of CH-EP had a cleaning frequency of less than a month, only 13% of AR-EP showed this cleaning frequency, while the rest cleaned the pens at the end of each breeding period or never. Differences between the two countries were not statistically significant (*p* = 0.0877).

**Figure 4:**
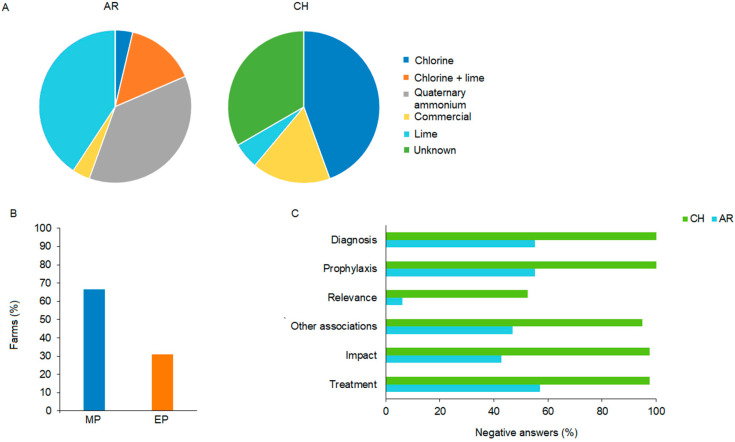
Disinfectants, chemical drug usage, and knowledge of chicken coccidiosis. (**A**) Distribution of the types of disinfectants used in FPPS in AR and CH. (**B**) Percentages of Argentinian meat (MP) and egg-productive (EP) farms that use chemical drugs. (**C**) Percentages of negative answers regarding diagnosis, prophylaxis, relevance, the impact, and treatment of coccidiosis in FPPSs in Argentina (AR) and Chile (CH). The survey included closed questions (yes or no).

Regarding the usage of disinfectants, 49.4% of family producers used disinfectants and 50.6% did not, with no statistically significant differences between the two countries (*p* = 0.9999). It was observed that 42.5 and 45.7% of family producers in Argentina and Chile, respectively, used disinfectants on their farms, the most used being lime in Argentina (45.8%) and chlorine in Chile (44.4%) (Figure 4A).

### 3.5. Use of Chemicals and Anticoccidials

The survey indicated a low level of chemical use in both countries. It showed that 69.0 of AR-MP and 73.3% of CH-EP did not use chemicals, and no significant difference (*p* = 0.2321) was found. Importantly, when comparing Argentinian production scenarios, it was found that 66.7% of AR-MP used chemical drugs, such as antibiotics, parasiticides, and anticoccidials, while 31.0% of AR-EP also used chemical drugs, which marked a statistically significant difference (*p* = 0.0303) (Figure 4B; Table S1). Specifically, sulfonamides, used as anticoccidials, were employed in all but one clinical case in broilers at the breeding stage in Argentina.

### 3.6. Knowledge of the Disease

Questions were asked about knowledge of the disease, its impact on production, and control measures adopted by family producers in both countries. The results showed that 58.4% of the producers were unaware of the disease, with a significant difference (*p* = 0.0017) between countries (30.6 vs. 92.5% unawareness for Argentina and Chile, respectively). In Chile, 97.5% of producers did not know about the impact of chicken coccidiosis or its association with other diseases. Consequently, 100% did not carry out diagnosis, prophylaxis, or treatment. In contrast, 43% of Argentinian producers were unaware (43%) of its impact, meaning most of the surveyed Argentine family producers were aware of the effects of coccidiosis (57.0%) and its association with other diseases (53.0%). However, more than half had not carried out diagnosis (55.1%), prophylaxis (55.1%), or treatment (57.1%), nor had they observed signs of coccidiosis (Figure 4C; Table S1).

## 4. Discussion

Poultry production is one of the most developed agricultural activities in family farming and plays a fundamental role in providing quality, healthy, and safe food, contributing to food security worldwide. Family poultry farming in low- and middle-income countries occurs in rural, urban, and peri-urban areas and encompasses various small-scale production systems. Of the four major categories distinguished by the FAO [2], three were observed in the studied areas of Argentina and Chile: small-scale extensive scavenging, semi-intensive, and small-scale intensive. Small-scale extensive systems were not included in this study as, in these cases, breeding is used for self-sufficiency without market access. In Argentina, 25% of the farms investigated were dedicated to meat, 65% to eggs, and 5% to mixed production, while in Chile 100% were dedicated to egg production. The lower percentage of MP vs. EP in Argentina derives from the fact that only a few slaughterhouses are authorized by Argentine state agencies for establishments with small production. Of note, government programs are being implemented to ensure that home-based work is safe and harmless through good slaughter practice workshops, with the implementation of an economic prototype developed by the INTA, particularly focused on female farmers [22].

Control strategies for avian coccidiosis are mainly addressed to intensive production systems, and there are no data on the prevalence of *Eimeria* infections in the FPPSs of Argentina and Chile. Thus, assessing the epidemiological situation of coccidiosis in this region is of the utmost importance to design strategies for improving chicken health and productivity and diminishing the environmental impact of chemical anticoccidials. The high overall positivity rates observed in the present study are in line with previous reports from commercial flocks in Argentina (80.0%) and Chile (76.5%) [23,24]. However, the reported prevalence of coccidiosis in backyard systems in different parts of the world was highly variable, ranging from 25.8 to 85.7% [8,10,11,25,26]. In the present study, an overall prevalence of 84.2% was found, which is similar to rates found in Greek production systems (87.7%) and Australian backyard flocks (81.0%) [8,9,10,11]. This highlights the high levels of *Eimeria* transit and consequent environmental oocyst dissemination in chicken farms. The persistence of *Eimeria* species in FPPSs may act as a reservoir, as indicated by other authors [8,11]. Therefore, it is necessary to understand the local interrelationships between the different types of production systems. In this sense, interventions have been carried out in Chile to avoid the risk of transmission of diseases from FPPSs to commercial flocks [27].

The dissemination of *Eimerian*-oocysts through the environment can be particularly threatening for industrial-scale chicken production and breeding centers where coccidiosis can cause high mortality. Recently, the prevalence of coccidiosis and ascaridiosis was predicted in different low- and middle-income countries, including Argentina [28]. Using regression imputation methods, prevalence rates of 23% and 12% were predicted for Argentina and the province of Buenos Aires, respectively. In the present study, we observed seven-times-higher rates, which could be explained by the fact that the prediction model only considered climatic factors from 2001 and 2021, not considering other factors like animal husbandry management practices, which proved to be very variable, the age of the birds, or the chicken lines. Another limitation of the model is that no data on the historical prevalence of coccidiosis could be used since they were not available for Argentinian and Chilean backyard systems. Thus, the present study could provide additional data to enhance the power of predictive models in future studies.

When comparing OPG levels according to the rearing stage for each type of backyard production, high rather than low levels were found in broilers at the breeding and rebreeding stages (up to 45 days of age), but this was not observed in hens at the same production stages (up to 60 days of age). This suggests that factors other than age, which were not studied here, may influence *Eimeria* infection. Moreover, the high proportion of low levels found in older chickens indicates an environmental spread, emphasizing the importance of separating young birds from adults, a practice often observed in FPPS rearing, especially in Chile.

Differences in coccidiosis clinical signs observed in broilers and laying hens are consistent with the evolution of the disease, where young animals are more susceptible as their immune system is not completely developed until after a few weeks of age, and older chickens acquire immunity after successive re-exposure to the parasite [10,29]. The distribution of *Eimerian*-chicken species is variable worldwide and differs between types of production and geographical regions. We present here the first data obtained for Chilean FPPSs, and in the case of Argentina, the first obtained by molecular methodologies. For the latter, *Eimeria* spp. frequencies are consistent with those previously found for commercial farms in Argentina using traditional parasitology methods [30]. Importantly, the most common species found, i.e., *E. mitis* (70.3%), *E. acervulina* (62.2%), *E. tenella* (59.5%), and *E. maxima* (43.2%), were also found in the oldest asymptomatic chickens, which may be spreading the parasite. Mixed infections are commonly found in chicken coccidiosis, and in the present study, combinations of two and three species were the most frequently observed. This agrees well with observations in backyard chicken farms in Greece and North India, where mixed infections were much more common than single infections, but is in contrast with data from alternative poultry production systems in Brazil, which showed that single infections were the most frequent [12].

Interestingly, six out of eight single infections in EP contained *E. mitis*. Although this is a less commonly reported species worldwide, *E. mitis* was found to have a 54% prevalence in Australian backyard production systems [8], 38% in alternative poultry production systems in Brazil [12], 40% in North India [31], and 38.9% in South Africa [14]. Importantly, these data were obtained using different molecular protocols, and thus, the percentages may be non-comparable. Molecular assays used to identify the seven chicken *Eimeria* sp. include single or nested PCR based on genetic markers internal transcribed spacers (1 or 2), 5.8S or 28S ribosomal DNA sequences, and multiplex PCR based on SCAR sequences [5]. We have chosen and optimized the latter method given its simplicity, simultaneity, and specificity [20,21], as confirmed by NGT sequencing in the current study. Altogether, the molecular results suggest that the distribution of *Eimeria* spp. may vary geographically and between production systems, highlighting the need to better understand the impact of coccidiosis and the relationship among the different poultry production systems.

Coccidiosis infections can be clinical or subclinical. The former is generally characterized by diarrhea of variable intensity, including bloody diarrhea, sometimes accompanied by secondary bacterial infections and, in serious cases, death. Subclinical forms manifest with weight loss, which can be observed as an uneven chicken size and decreased egg production. *Eimeria* spp. possess different levels of pathogenicity; hemorrhagic forms are caused by *E. brunetti*, *E. necatrix*, and *E. tenella,* and species that cause malabsorption are *E. acervulina*, *E. maxima*, *E. mitis*, and *E. praecox* [32]. Although chickens may recover from coccidiosis, their productivity levels are unlikely to return to pre-infection levels [33]. We found infections with mixtures of two, three, or six species in clinical and subclinical cases. *E. tenella* or *E. brunetti* was found in the majority (16/21) of samples with bloody diarrhea, but they were also found in asymptomatic cases (6/14), highlighting the importance of molecular diagnosis to implement control measures even in asymptomatic chickens.

Although poor cleaning and inefficient disinfection are not the only influencing factors, they may explain the high prevalence of *Eimeria* spp. found in this study by allowing oocysts to persist in the environment. Biosecurity measures are key to controlling diseases such as coccidiosis and should be improved by increasing the frequency of cleaning, and by using effective disinfectants in the sheds. In the surveys, it was observed that lime is the disinfectant most commonly used in Argentinian farms and chlorine in Chile, but none of the disinfectants are reported to be effective against *Eimeria* sp. [34,35]. Effective compounds against *Eimeria*-oocysts are ammonium hydroxide and ammonia, carbon disulfide compounds, and hydrogen peroxide if applied at high concentrations, and it has been also shown that ozone treatment inactivates oocysts [35]. This is especially important for semi-intensive systems where chickens are kept in sheds in the evening and during the night.

In addition to biosecurity measures and good animal husbandry management practices, clinical coccidiosis in FPPSs is controlled by the use of anticoccidials, especially in AR-MP (Figure 4B). The majority of EP farms did not use anticoccidials (69.0 and 73.3% of AR-MP and CH-EP, respectively); therefore, in the studied region, egg-productive farms do not possess prior history or treatment. In contrast, 66.7% of AR-MP used chemical drugs, such as antibiotics, parasiticides, and anticoccidials, with sulfonamides used in all but one clinical case. It interesting to highlight that international organizations have warned of these chemicals due to their environmental damage, emergence of resistant strains, and public health risks, highlighting the urgent need for cost-effective and safe tools to alleviate coccidiosis [4,36,37,38], to diminish their environmental impact and improve food safety.

In Chile, 97.5% of producers did not know about the impact of chicken coccidiosis or its association with other diseases. Consequently, 100% did not use prophylaxis, treatment, or diagnosis. These results align with previous reports that Chilean farmers did not know how to identify the diseases affecting their chickens and lacked control measures for coccidiosis in backyard poultry production [1,9]. In contrast, INTA-Argentina veterinarians provide advice and productive support to farmers, which shows the importance of training in preventing poultry diseases. Although most of the surveyed Argentine family producers were aware of the effects of coccidiosis and its association with other diseases, less than half had carried out prophylaxis, diagnosis, and treatment (Figure 4C), highlighting the need for more formal training. Implementation of affordable measures for control of this complex parasitic disease in family poultry is of special interest, given that most farms do not have the resources to apply live vaccination. This would especially benefit women farmers, who are mainly dedicated to chicken production [39].

The current cross-sectional study was carried out in peri-urban areas of the Metropolitan Area of Buenos Aires, Argentina; Province of Buenos Aires, Argentina; Santiago Metropolitan Region, Chile; and Libertador General Bernardo O’Higgins Region, Chile, and focused on small farms that commercialized their products (*n* = 88). This observational study analyzed data to help establish preliminary evidence for future studies addressing the introduction of chicken coccidiosis control measures in FPPSs, which will improve their productivity. Moreover, traditional and molecular techniques allowed us to establish the *Eimeria* prevalence and the chicken species distribution. Some limitations of the cross-sectional design in survey research [40] can be mentioned, such as the temporal ambiguity—since data are collected at a single point in time, it is difficult to establish the temporal order of events, which is crucial for inferring causality; lack of control over variables, which makes it challenging to isolate the effect of the independent variable on the dependent variable; and retrospective reporting issues—asking respondents to recall past events or changes can introduce recall bias and inaccuracies in the data, especially when the level of awareness is very low, as was demonstrated in the current surveys. Overall, while the current cross-sectional study provides valuable insights on chicken coccidiosis in FPPSs in Argentina and Chile, it has limitations in establishing causal relationships. However, this study could be a starting point for establishing epidemiological surveillance and measuring changes over time.

## 5. Conclusions

This work is the first approximation of the characterization of FPPSs for the region and provides insight into the current situation of chicken coccidiosis and the diversity of *Eimeria* species circulating in Argentina and Chile. The limited knowledge of the disease and the lack of effective control measures, combined with the high prevalence and diversity of *Eimeria* spp. found in the current work, indicate that FPPSs in both South American countries contribute to the spread of parasites, including pathogenic *Eimeria* spp., and that they also contribute to environmental pollution by sulfonamides in the case of Argentina. The knowledge gained is useful for introducing hygiene improvements that will not only reduce the environmental spread of *Eimeria* but also decrease the use of harmful anticoccidials.

More in-depth studies are needed to better understand the relationship between FPPSs and industrial poultry, by increasing sampling, covering more regions, and identifying new *Eimeria* genetic variants not previously studied in these countries.

## Data Availability

DNA sequences were deposited in Genbank under accession numbers PP328761–PP328774.

## Supplementary Materials

The following supporting information can be downloaded at https://www.mdpi.com/article/10.3390/ani15070982/s1, Table S1. Summary of data obtained for FPPSs in Argentina and Chile regarding types of production, cleaning and disinfection strategies, *Eimeria* sp. oocyst loads, and farmers’ knowledge od coccidiosis.

## Abbreviations

The following abbreviations are used in this manuscript:

AMBA	Metropolitan Area of Buenos Aires
AR	Argentina
BA	Buenos Aires Province
CH	Chile
dNTP	Deoxy-Nucleotide Triphosphate
EP	Egg Production Farm
FPPS	Family Poultry Production System
INTA	National Institute of Agriculture Technology
MP	Meat Production Farm
OH	Libertador General Bernardo O’Higgins Region
OPG	Oocysts per Gram
PCR	Polymerase Chain Reaction
RM	Santiago Metropolitan Region
SCAR	Sequence-Characterized Amplified Region
