# Supplementary material for: Chicken Coccidiosis in Peri-Urban Family Farming in Two South American Countries: Prevalence and Circulating Eimeria spp
Source: Animals (Basel). 2025 Mar 29;15(7):982. doi: 10.3390/ani15070982 (PMC11987751; doi:10.3390/ani15070982)
Supplement: Supplementary file 1 [file animals-15-00982-s001.zip › animals-3460058-supplementary.pdf]

Table S1. Summary of data obtained for FPPS in Argentina and Chile regarding types of production, cleaning and disinfection strategies, *Eimeria* sp. oocyst loads and farmers´ knowledge on coccidiosis

				Cleaning and disinfection				Coccidiosis									
	Region	Type of production	Category	Cleaning	Disinfectant	Type of disinfectant	Use of drugs	Knowledge of the coccidiosis	Knowledge of the impact	Association with other diseases	Prophylaxis	Diagnosis	Treatment	Diarrhea	UnS	OPG	OPG Level
1	AMBA	MP	s-i	Er	No		S	Yes	Yes	Yes	No	Yes	Yes	Yes	Yes	22,946	H
2	AMBA	MP	s-i	Er	No		Ac	Yes	Yes	Yes	No	Yes	Yes	Yes	No	322	L
3	AMBA	MP	Si	Er	Yes	Lime	No	Yes	Yes	No	No	Yes	Yes	No	Yes	20,440	H
4	AMBA	MP	s-i	Er	No		S	Yes	Yes	Yes	No	Yes	Yes	Yes	No	13,230	H
5	AMBA	MP	Si	Er	Yes	Qa	No	Yes	Yes	Yes	Yes	Yes	Yes	Yes	No	17,122	H
6	AMBA	MP	s-i	Er	No		No	Yes	Yes	Yes	Yes	Yes	Yes	No	Yes	6,356	H
7	AMBA	EP	s-i	Lm	Yes	Qa	NI	Yes	Yes	Yes	Yes	Yes	Yes	No	Yes	0	
8	AMBA	EP	s-i	Er	Yes	Lime	No	No	No	No	No	No	No	No	Yes	0	
9	AMBA	EP	Si	Er	Yes	Qa	No	Yes	No	Yes	Yes	Yes	Yes	Yes	Yes	0	
10	AMBA	EP	Si	Ue	No		Ap	No	No	No	No	No	No	Yes	Yes	224	L
11	AMBA	MP	E	Ue	No		S	Yes	Yes	Yes	Yes	Yes	Yes	Yes	Yes	150	L
12	AMBA	EP	Si	Ue	No		S	Yes	Yes	Yes	Yes	Yes	Yes	No	Yes	210	L
13	AMBA	MP	Si	Lm	No		S	Yes	Yes	Yes	Yes	Yes	Yes	Yes	Yes	3,390	M
14	AMBA	MP	Si	Ue	No		Ab	Yes	No	Yes	No	Yes	Yes	Yes	Yes	1,120	L
15	AMBA	EP	s-i	Lm	No		S, Ab	Yes	Yes	Yes	Yes	Yes	Yes	No	Yes	1,400	L
16	AMBA	EP	s-i	Ue	NI		Ab	Yes	Yes	Yes	Yes	Yes	Yes	No	Yes	532	L
17	AMBA	EP	Si	Er	No		S, Ab	Yes	Yes	No	No	Yes	Yes	Yes	Yes	2,212	M
18	AMBA	MP	E	Er	Yes	Qa	No	Yes	Yes	No	Yes	Yes	Yes	Yes	No	150	L
19	AMBA	EP	Si	Er	No		No	Yes	Yes	No	No	No	No	Yes	No	952	L
20	AMBA	EP	E	Er	No		No	Yes	No	No	No	No	No	Yes	No	357	L
21	AMBA	MP	Si	Er	No		Ac	Yes	Yes	Yes	Yes	Yes	Yes	Yes	Yes	1,099	L
22	BA	MP	E	Er	No		No	Yes	Yes	Yes	Yes	Yes	Yes	Yes	No	7,826	H
23	BA	EP	E	Lm	Yes	CL	No	No	No	No	No	No	No	Yes	No	294	L
24	BA	EP	s-i	Er	Yes	Lime	No	No	No	No	No	No	No	No	No	0	
25	BA	EP	s-i	Er	No		No	Yes	Yes	No	Yes	Yes	Yes	No	No	0	
26	BA	EP/MP	s-i	Er	Natural		No	No	No	No	No	No	No	No	No	812/476	L/L
27	BA	EP	E	Ue	Yes	Lime	No	No	No	No	No	No	No	No	Yes	0	
28	BA	EP	s-i	Ue	Yes	Lime	No	No	No	No	No	No	No	No	No	126	L
29	BA	EP	s-i	Ue	Yes	Lime	No	No	No	No	No	No	No	No	Yes	294	L
30	BA	EP	Si	Er	No		No	Yes	Yes	Yes	Yes	No	No	No	No	97	L
31	BA	EP	s-i	Ue	No		No	Yes	Yes	Yes	Yes	No	No	No	No	752	L
32	BA	EP	s-i	Ue	No		No	No	No	No	No	No	No	No	No	511	L

33	BA	EP	E	Lm	No		No	No	No	No	No	No	No	No	No	728	L
34	BA	EP/MP	s-i	Ue	No		No	NI	NI	NI	NI	NI	NI	No	No	388/245	L/L
35	BA	EP	E	Er	Yes	Qa	NI	Yes	Yes	Yes	No	No	No	No	No	1431	L
36	BA	EP/MP	Si	Er	Yes	Qa	No	Yes	Yes	Yes	Yes	Yes	Yes	No	No	0/42	L
37	BA	EP	s-i	Ue	No		No	Yes	Yes	No	No	No	No	No	No	0	
38	BA	EP/MP	s-i	Ue	Yes	Lime	NI	Yes	Yes	No	No	No	No	No	Yes	63/4,807	L/M
39	BA	EP	Si	Ue	Yes	Qa	No	Yes	No	No	No	No	No	Yes	No	640	L
40	BA	EP	E	Ue	Yes	Lime	No	No	No	No	No	No	No	Yes	No	574	L
41	BA	EP	s-i	Er	Yes	Commercial	No	Yes	Yes	No	No	No	No	No	No	171	L
42	BA	EP/MP	s-i	Er	Yes	CL	No	No	No	No	No	No	No	No	No	168/7,825	L/H
43	BA	EP	Si	Er	Yes	CL	S, Ap	Yes	No	No	Yes	No	No	No	No	77	L
44	BA	EP	E	Er	Yes	Lime	Ap, Ab	No	No	No	Yes	No	No	No	No	280	L
45	BA	EP	E	Er	No		Ap, Ab	No	No	No	Yes	No	No	No	No	0	
46	BA	EP	E	Er	Yes	CL, Qa	Ap	No	No	No	Yes	No	No	No	No	112	L
47	BA	EP	Si	Er	Yes	Chlorine	No	Yes	Yes	Yes	Yes	No	No	No	No	227	L
48	BA	EP	Si	Er	Yes	Lime, Qa	No	Yes	Yes	Yes	No	Yes	No	No	No	0	
49	BA	EP	Si	Er	Yes	Lime, Qa	No	Yes	Yes	Yes	No	No	No	No	No	70	L
50	RM	EP	s-i	Lm	Yes	Lime	NI	No	No	No	No	No	No	No	No	192	L
51	RM	EP	Si	Lm	NI		Ab	Yes	Yes	Yes	No	No	Yes	No	No	203	L
52	RM	EP	s-i	Lm	Yes	Unknown	NI	No	No	No	No	No	No	No	No	118	L
53	RM	EP	s-i	Lm	Yes	Chlorine	NI	No	No	No	No	No	No	No	No	25	L
54	RM	EP	s-i	Er	Yes	Chlorine	NI	No	No	No	No	No	No	No	No	77	L
55	RM	EP	Si	Lm	Yes	Chlorine	NI	No	No	No	No	No	No	No	No	49	L
56	RM	EP	s-i	Lm	Yes	Chlorine	NI	No	No	No	No	No	No	No	No	33	L
57	RM	EP	Si	Er	Yes	Chlorine	NI	No	No	No	No	No	No	No	No	63	L
58	OH	EP	Si	Lm	Yes	Commercial	Ab	No	No	No	No	No	No	No	No	0	
59	OH	EP	Si	Lm	No		No	No	No	No	No	No	No	No	No	39	L
60	OH	EP	s-i	Lm	No		No	No	No	No	No	No	No	No	No	1,226	L
61	OH	EP	Si	Ue	No		No	No	No	No	No	No	No	No	No	21	L
62	OH	EP	Si	Lm	No		No	No	No	No	No	No	No	No	No	294	L
63	OH	EP	s-i	Lm	NI	Unknown	NI	No	No	No	No	No	No	No	No	56	L
64	OH	EP	Si	Lm	Yes	Unknown	NI	No	No	No	No	No	No	Yes	Ni	0	
65	OH	EP	Si	Lm	Yes	Unknown	NI	No	No	No	No	No	No	No	No	70	L
66	OH	EP	Si	Lm	Yes	Commercial	NI	No	No	No	No	No	No	No	No	0	
67	OH	EP	Si	Lm	Natural		Natural	No	No	No	No	No	No	No	No	25	L
68	RM	EP	E	Ue	No	Commercial	Ap	No	No	No	No	No	No	No	Yes	425	L
69	RM	EP	s-i	Ue	No		No	No	No	No	No	No	No	No	No	50	L
70	RM	EP	E	Ue	Yes	Chlorine	Ap, Ab	No	No	Yes	No	No	No	No	No	0	

71	RM	EP	E	Ue	Yes	Chlorine	Ap	No	No	No	No	No	No	No	No	250	L
72	RM	EP	E	Ue	No		Ap	No	No	No	No	No	No	No	No	5,625	M
73	RM	EP	s-i	Ue	No		No	Yes	No	No	No	No	No	No	No	175	L
74	RM	EP	s-i	Ue	Yes	Chlorine	Ap	Yes	No	No	No	No	No	No	No	375	L
75	OH	EP	s-i	N	No		No	No	No	No	No	No	No	NI	NI	25	L
76	OH	EP	s-i	Ue	Yes	Unknown	No	No	No	No	No	No	No	NI	NI	200	L
77	OH	EP	Si	Ue	Yes	Unknown	No	No	No	No	No	No	No	NI	NI	250	L
78	OH	EP	E	N	No		No	No	No	No	No	No	No	NI	NI	100	L
79	RM	EP	s-i	N	No		No	No	No	No	No	No	No	NI	NI	1,525	L
80	RM	EP	E	N	No		No	No	No	No	No	No	No	NI	NI	9,225	H
81	RM	EP	s-i	N	No		No	No	No	No	No	No	No	NI	NI	725	L
82	RM	EP	E	N	No		No	No	No	No	No	No	No	NI	NI	1,175	L
83	RM	EP	s-i	Ue	Natural		No	No	No	No	No	No	No	NI	NI	225	L
84	RM	EP	s-i	N	No		No	No	No	No	No	No	No	NI	NI	200	L
85	OH	EP	s-i	N	No		No	No	No	No	No	No	No	NI	NI	105	L
86	OH	EP	s-i	N	No		No	No	No	No	No	No	No	NI	NI	8,375	H
87	OH	EP	E	N	No		No	No	No	No	No	No	No	NI	NI	1,250	L
88	OH	EP	E	N	No		No	No	No	No	No	No	No	NI	NI	125	L

Ab: Antibiotics; Ac: Anticoccidial; AMBA: Metropolitan Area of Buenos Aires, Argentina; Ap: Antiparasitics; BA: Province of Buenos Aires, Argentina; Qa: quaternary ammonium; CL: chlorine and lime; E: Extensive; EP: egg production; Er: End of the rearing; H: high; L: low; Lm: Less than a month; M: medium; MP: meat production; N: never; Natural: ashes or white vinegar; NI: not informed; OH: Libertador General Bernardo O'Higgins Region, Chile; RM. Santiago Metropolitan Region, Chile; S: Sulfonamides; Si: Small intensive; s-i: semi-intensive; Ue: Unwanted events; UnS: uneven chicken size.
